# Protective Effects of Vitamin D Against Doxorubicin Chemotherapy–Induced Hepatotoxicity in Wistar Albino Rats: Evidence from ^99^mTc-Pyrophosphate Scintigraphy and Oxidative–Inflammatory Pathways

**DOI:** 10.3390/nu18071097

**Published:** 2026-03-29

**Authors:** Murat Kalın, Haluk Kerim Karakullukcu, Mina Karakullukcu, Aylin Arslan, Serdar Savaş Gül, Reyhan Toyran, Ömer Faruk Özkan, Gülçin Ercan, Hatice Aygun

**Affiliations:** 1 Department of General Surgery, Sultan 2. Abdulhamid Han Educational and Research Hospital, University of Health Sciences, Istanbul 34668, Turkey; murat.kalin@hotmail.com (M.K.); halukkarakullukcu@gmail.com (H.K.K.); omerfaruk.ozkan@sbu.edu.tr (Ö.F.Ö.); gulcin.ercan@sbu.edu.tr (G.E.); 2 Department of Oncology, Sultan 2. Abdulhamid Han Educational and Research Hospital, University of Health Sciences, Istanbul 34668, Turkey; mina.zoralioglu@gmail.com; 3 Department of General Surgery, Istanbul Prof. Dr. Cemil Taşçıoğlu City Hospital, University of Health Sciences, Istanbul 34668, Turkey; aylinarslan53@outlook.com; 4 Department of Nuclear Medicine, Faculty of Medicine, Lokman Hekim University, Ankara 06530, Turkey; serdar.gul@lokmanhekim.edu.tr; 5 Department of Nuclear Medicine, Istanbul Sultan 2. Abdulhamid Han Training and Research Hospital, İstanbul 34668, Turkey; reyhankoroglu@yahoo.com; 6 Neuroscience Laboratory, BAMER, Biruni University, Istanbul 34010, Turkey

**Keywords:** doxorubicin hepatotoxicity, vitamin D, ^99^mTc-PYP scintigraphy, oxidative stress, inflammation

## Abstract

**Objectives:** Doxorubicin, a widely used chemotherapeutic agent, is known to induce hepatotoxicity through oxidative stress and inflammatory pathways. Vitamin D has been reported to exert antioxidant and immunomodulatory effects; however, its potential protective role in doxorubicin-induced liver injury remains insufficiently characterized. **Materials and Methods:** Adult male Wistar albino rats were randomly assigned to six groups (*n* = 7): Control, Vitamin D (5000 IU/kg), Vitamin D (60,000 IU/kg), Doxorubicin, DOX + Vitamin D (5000 IU/kg), and DOX + Vitamin D (60,000 IU/kg). Vitamin D_3_ (cholecalciferol) was administered orally either as a daily dose (5000 IU/kg for 12 days) or as a single bolus dose (60,000 IU/kg). Doxorubicin (6 mg/kg/day, cumulative dose 18 mg/kg) was administered intraperitoneally on days 10–12. Hepatic injury was evaluated using ^99^mTc-pyrophosphate (^99^mTc-PYP) scintigraphy, serum liver enzymes (AST, ALT, LDH, total bilirubin), renal markers (BUN, creatinine), calcium and 25-hydroxyvitamin D [25(OH)D], oxidative stress parameters (MDA, TOS, TAS, GSH, SOD, Nrf2), and inflammatory cytokines (TNF-α, IL-6, IL-1β, IL-10). **Results:** Doxorubicin markedly increased hepatic ^99^mTc-PYP uptake and significantly elevated AST, ALT, LDH, bilirubin, MDA, TOS, TNF-α, IL-6, and IL-1β levels while reducing Nrf2, GSH, SOD, TAS, and IL-10 (all *p* < 0.001). Vitamin D supplementation significantly increased serum 25-hydroxyvitamin D [25(OH)D] levels compared with controls (32.3 ± 2.7 vs. 74.1 ± 3.8 and 69.3 ± 3.2 ng/mL for the 5000 and 60,000 IU/kg groups, respectively; *p* < 0.001) and attenuated DOX-induced hepatic injury, as indicated by reduced radiotracer uptake and improved oxidative and inflammatory markers. Vitamin D also mitigated DOX-associated increases in renal injury markers (BUN and creatinine) without inducing hypercalcemia. No significant differences were observed between the two vitamin D dosing regimens in most outcome measures. **Conclusion:** Vitamin D supplementation exerted protective effects against doxorubicin-induced liver injury, likely through modulation of oxidative stress and inflammatory pathways. Additionally, ^99^mTc-PYP scintigraphy may serve as a useful imaging tool for detecting acute hepatocellular injury and evaluating therapeutic responses.

## 1. Introduction

Cancer remains a major global health problem, with 19.3 million new cases and 10 million deaths reported in 2020 [1]. Chemotherapy is a key component of cancer treatment, with doxorubicin (DOX) widely used for breast cancer, lymphomas, sarcomas, and several solid tumors [2,3]. However, its use is limited by systemic toxicities, including cardiotoxicity, nephrotoxicity, and hepatotoxicity [4,5,6]. Although liver injury is often overlooked compared to cardiac damage, several preclinical studies confirm that DOX leads to acute hepatocellular injury [7,8,9].

DOX induces oxidative stress by generating reactive oxygen species (ROS), which damage lipids, proteins, and DNA [10]. This is accompanied by inflammation via activation of NF-κB and cytokine release [11]. Together, oxidative stress and inflammation form a central mechanism of DOX-induced organ toxicity.

Vitamin D exerts biological effects beyond calcium and bone metabolism through activation of the vitamin D receptor (VDR). VDR is widely expressed across tissues, including the liver, where it is primarily localized in non-parenchymal cells such as Kupffer cells, sinusoidal endothelial cells, and hepatic stellate cells [12,13,14]. This distribution indicates that vitamin D signaling may play a role in regulating hepatic immune responses and fibrogenic processes rather than serving solely endocrine skeletal functions. Recent research shows that vitamin D has antioxidant and anti-inflammatory roles by upregulating protective enzymes and inhibiting pro-inflammatory cytokines [15]. In liver disease models, vitamin D has shown antifibrotic and hepatoprotective effects [16,17]. Despite its therapeutic potential, experimental data on vitamin D’s role in doxorubicin-induced hepatotoxicity remain scarce [18].

This study evaluates the protective effects of vitamin D against acute DOX-induced liver injury using two regimens: 5000 IU/kg daily and a single 60,000 IU/kg bolus. In addition, ^99^mTc-pyrophosphate (^99^mTc-PYP) scintigraphy was used to detect acute hepatic injury. Although ^99^mTc-PYP has shown uptake in necrotic liver tissue [19], no animal study has applied this method to evaluate DOX-related hepatotoxicity. We investigated the hepatoprotective effects of vitamin D at different doses against DOX-induced acute liver injury and assessed the potential of ^99^mTc-PYP imaging as a non-invasive marker of hepatic damage and biochemical parameters.

## 2. Materials and Methods

### 2.1. Animal

A total of 42 adult male Wistar–Albino rats weighing 260–300 g (approximately 10–12 weeks old) were used in the study. Animals were allowed to acclimatize to the animal facility for one week before the start of the experiment. Rats were housed in standard cages under controlled environmental conditions (22–25 °C, 12 h light/12 h dark cycle). All experimental procedures were performed daily between 09:00 and 10:00 a.m. to maintain circadian rhythm consistency. Body weights were recorded at the beginning and at the end of the experimental period.

Rats had ad libitum access to standard pelleted rodent chow (Rat and Mouse No. 3 Breeding Diet, RM3 (P), product code 801700; Special Diets Services, SDS^®^, Essex, UK; distributed locally by Kobay Yem Sanayi, Ankara, Türkiye) and water. The vitamin D_3_ content of the diet was approximately 2900 IU/kg. Typical chow intake in rats is approximately 6–8 g per 100 g body weight per day. Based on this consumption rate, the estimated dietary vitamin D intake in rats weighing 260–300 g corresponds to approximately 45–70 IU/day (≈170–230 IU/kg/day). Thus, dietary vitamin D intake was minimal compared with the pharmacological vitamin D doses used in the experimental groups.

Throughout the study, animals were monitored at least once daily for changes in body weight, behavior, activity, posture, food, and water intake, and general clinical condition. When necessary, veterinary consultation was obtained to ensure animal welfare.

All experimental procedures were conducted in accordance with humane animal care guidelines and were approved by the Tokat Gaziosmanpasa University Animal Experiments Local Ethics Committee (Approval No: HADYEK 2017-16).

### 2.2. Drug Administration

Vitamin D_3_ (cholecalciferol) was administered by oral gavage using an oil-based formulation containing refined sunflower oil as the vehicle. The administered volume was adjusted according to body weight and ranged from approximately 0.4 to 0.6 mL for rats weighing 260–300 g (mean ≈ 0.5 mL). Control animals received an equivalent volume of refined sunflower oil as the vehicle.

To investigate the biological effects of vitamin D administered as a single high-dose bolus versus repeated daily dosing, two supplementation regimens were used. A high-dose bolus group received vitamin D_3_ (60,000 IU/kg) as a single oral administration, whereas a daily supplementation group received vitamin D_3_ (5000 IU/kg/day) for 12 consecutive days. These doses were selected based on previously published experimental studies evaluating pharmacological vitamin D supplementation in rodent models [20,21].

Doxorubicin hydrochloride (Sigma-Aldrich, St. Louis, MO, USA) was dissolved in sterile physiological saline (0.9% NaCl) and administered intraperitoneally during the last three days of the experimental protocol (days 10–12) at 6 mg/kg/day, resulting in a cumulative dose of 18 mg/kg over three days [22]. All administered drug doses were calculated according to individual body weight. Oral administrations were performed using a stainless-steel gavage needle, and all injections were carried out using sterile disposable syringes.

### 2.3. Experimental Procedure

A total of 42 rats were randomly allocated into six experimental groups (*n* = 7 per group).

Group I (Control):

Rats received refined sunflower oil by oral gavage (≈0.5 mL) for 12 consecutive days. During the last three days of the experimental protocol (days 10–12), animals received intraperitoneal injections of physiological saline (approximately 1 mL injection volume).

Group II (Vitamin D 5000 IU/kg):

Rats received vitamin D_3_ (cholecalciferol) at a dose of 5000 IU/kg/day by oral gavage (≈0.5 mL) for 12 consecutive days. During days 10–12, animals received intraperitoneal saline injections (approximately 1 mL).

Group III (Vitamin D 60,000 IU/kg):

Rats received a single oral bolus dose of vitamin D_3_ (60,000 IU/kg; ≈0.5 mL) on day 1. For the remaining days of the experimental protocol, animals received sunflower oil by oral gavage (≈0.5 mL) to maintain identical handling conditions. During days 10–12, animals received intraperitoneal saline injections (approximately 1 mL).

Group IV (DOX):

Rats received sunflower oil by oral gavage (≈0.5 mL) for 12 consecutive days, while doxorubicin was administered intraperitoneally during days 10–12 (6 mg/kg/day; cumulative dose 18 mg/kg; approximately 1 mL injection volume).

Group V (DOX + Vitamin D 5000 IU/kg):

Rats received vitamin D_3_ (5000 IU/kg/day; ≈0.5 mL) by oral gavage for 12 consecutive days, and doxorubicin was administered intraperitoneally during days 10–12 (6 mg/kg/day; approximately 1 mL).

Group VI (DOX + Vitamin D 60,000 IU/kg):

Rats received a single oral bolus dose of vitamin D_3_ (60,000 IU/kg; ≈0.5 mL) on day 1, followed by sunflower oil administration by oral gavage (≈0.5 mL) for the remaining days of the experimental protocol. Doxorubicin was administered intraperitoneally during days 10–12 (6 mg/kg/day; approximately 1 mL).

Subsequently, anesthesia was induced using ketamine (Ketalar^®^, 75 mg/kg; Pfizer, Istanbul, Türkiye) in combination with xylazine (Rompun^®^, 10 mg/kg; Bayer, Istanbul, Türkiye). Following adequate anesthesia, scintigraphic imaging was performed.

After imaging, blood samples were collected via cardiac puncture, centrifuged to obtain serum, and stored at −20 °C until analysis.

Immediately thereafter, liver tissues were excised, rapidly frozen, and stored at −80 °C for subsequent biochemical and molecular analyses.

### 2.4. ^99^mTc-Pyrophosphate (^99^mTc-PYP) Scintigraphic Imaging

On day 13 of the experimental protocol, anesthetized rats received 1 mCi of ^99^mTc-pyrophosphate (^99^mTc-PYP) via tail vein injection. One hour after tracer administration, static scintigraphic imaging was performed using a dual-head gamma camera (E.CAM, Siemens, Erlangen, Germany). Images were acquired in anterior and posterior projections with a zoom factor of 2.55. For semi-quantitative analysis, equal-sized rectangular regions of interest (ROIs) were manually drawn over the hepatic region. ROI placement and image analysis were performed by two blinded investigators to minimize observer bias.

### 2.5. Biochemical Assays

#### 2.5.1. Serum

At the end of the experimental period, all rats were anesthetized under light anesthesia, and blood samples were obtained via cardiac puncture into serum separator tubes. The collected samples were allowed to clot at room temperature for 30 min and were subsequently centrifuged at 1500× *g* for 10 min. The resulting clear serum was carefully separated and used for subsequent biochemical analyses.

Serum levels of aspartate aminotransferase (AST), alanine aminotransferase (ALT), lactate dehydrogenase (LDH), BUN, creatinine and total bilirubin were measured using kinetic assay methods with commercially available kits (Beckman Coulter, Brea, CA, USA). All analyses were performed on an automated biochemical analyzer (Beckman Coulter LX-2000, Brea, CA, USA) according to the manufacturer’s instructions. Serum total calcium levels were measured using an automated biochemical analyzer (Beckman Coulter LX-2000, Brea, CA, USA) based on the Arsenazo III colorimetric method according to the manufacturer’s instructions.

Serum 25-hydroxyvitamin D [25(OH)D] levels were determined by measuring 25-hydroxyvitamin D using a competitive ELISA kit (ELK Biotechnology, Wuhan, China; Cat. No. ELK0843) according to the manufacturer’s instructions. The assay detection range was 3.13–200 ng/mL with a sensitivity of 0.93 ng/mL, and absorbance was measured at 450 nm using a microplate reader.

Total antioxidant status (TAS) and total oxidant status (TOS) levels were determined using commercially available colorimetric assay kits (Rel Assay Diagnostics, Mega Tıp Sanayi ve Ticaret Ltd. Şti., Gaziantep, Turkey) according to the automated method developed by Erel. The TAS assay is based on the reduction of the ABTS radical cation by antioxidants present in the sample, and results are expressed as mmol Trolox equivalent per liter (mmol Trolox Eq/L). The TOS assay measures the oxidation of the ferrous ion–chelator complex to ferric ion in the presence of oxidant species, and the results are expressed as µmol hydrogen.

#### 2.5.2. Hepatic Biochemical Analysis

Hepatic levels of nuclear factor erythroid 2–related factor 2 (Nrf2), malondialdehyde (MDA), glutathione (GSH), superoxide dismutase (SOD), tumor necrosis factor-α (TNF-α), interleukin-6 (IL-6), interleukin-1β (IL-1β), interleukin-10 (IL-10), and high mobility group box-1 (HMGB1) were measured in liver tissue samples. All tissue specimens were washed with cold isotonic saline solution (0.9%), and wet tissue weights were recorded. Tissues were cut into small pieces, transferred into tubes, and homogenized in cold phosphate-buffered saline (PBS, pH 7.4) using stainless steel beads. Homogenates were centrifuged at 2500 rpm for 20 min, and the supernatants were collected for biochemical analyses. Quantification of tissue biomarkers was performed using enzyme-linked immunosorbent assay (ELISA) or colorimetric assay kits according to the manufacturers’ instructions.

Nrf2 levels in liver tissue homogenates were determined using a rat-specific ELISA kit (Rat NFE2L2 (Nrf2) ELISA Kit, Elabscience, Wuhan, China). MDA levels were measured using a rat-specific ELISA kit (Cat. No. MBS268427; MyBioSource, San Diego, CA, USA). GSH levels were analyzed using a commercial colorimetric assay kit (Cat. No. EEA020; Thermo Fisher Scientific, Invitrogen, Waltham, MA USA). Proinflammatory cytokines including IL-6, TNF-α, and IL-1β were quantified using rat-specific ELISA kits (Cat. Nos. E0135Ra, E0764Ra, and E0119Ra; Bioassay Technology Laboratory, Shanghai, China). Anti-inflammatory IL-10 levels were measured using a rat IL-10 ELISA kit (Cat. No. ERA23RB; Invitrogen, Thermo Fisher Scientific, Waltham, MA, USA). SOD activity was determined using a commercial assay kit (Item No. 706002; Cayman Chemical, Ann Arbor, MI, USA) and expressed as U/mg protein. HMGB1 levels were measured using an ELISA kit (Cat. No. ST51011; IBL International, Hamburg Germany).

### 2.6. Statistical Analysis

Normality assessment indicated that all variables were normally distributed across experimental groups, as confirmed by Kolmogorov–Smirnov and Shapiro–Wilk tests (all *p* > 0.05), supporting the use of parametric statistical analyses. Accordingly, group differences were evaluated using one-way analysis of variance (one-way ANOVA), followed by Tukey’s multiple-comparison test when appropriate. Body weight measurements obtained at multiple time points were analyzed using a two-way repeated measures analysis of variance (ANOVA), with time (Day 10–13) as the within-subject factor and treatment group as the between-subject factor. Post hoc comparisons were performed using Bonferroni correction to adjust for multiple comparisons and assess differences between groups at each time point. Data are presented as mean ± standard error of the mean (SEM).

## 3. Results

### 3.1. Body Weight Changes

**Figure 1:**
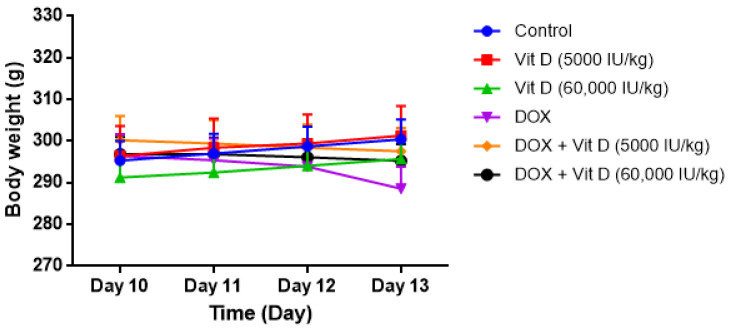
Effects of vitamin D on body weight in DOX-treated rats. Time-course of body weight changes from Day 10 to Day 13 across experimental groups. Data are presented as mean ± SEM. Two-way repeated measures ANOVA showed significant effects of time and time × group interaction, but no main effect of group; post hoc comparisons (Bonferroni) were not significant (*p* > 0.05).

Two-way repeated measures ANOVA revealed a significant effect of time (F(3,108) = 4.844, *p* = 0.0034) and a significant time × group interaction (F(15,108) = 50.68, *p* < 0.0001), while no main effect of group was observed (F(5,36) = 0.2279, *p* = 0.9480). Post hoc analysis using Bonferroni correction did not demonstrate significant intergroup differences at individual time points (all *p* > 0.05) (Figure 1).

Although DOX-treated animals exhibited a downward trend in body weight over time, vitamin D-treated groups showed a partial attenuation of this pattern; however, these differences did not reach statistical significance (Figure 1).

### 3.2. ^99^mTc-PYP Uptake in Liver

**Figure 2:**
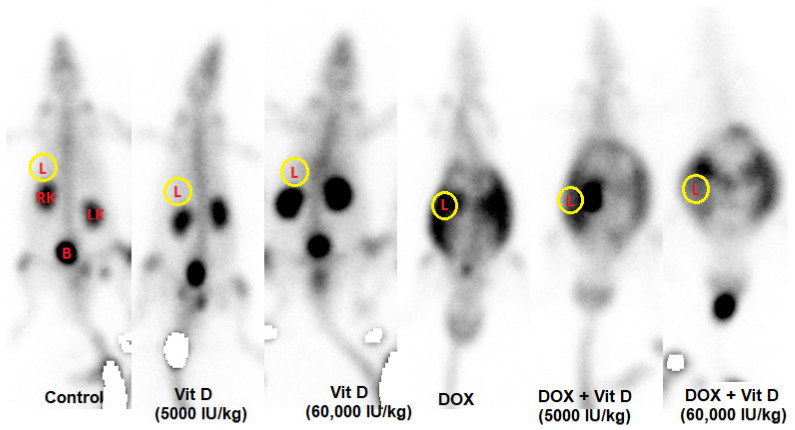
Representative whole-body scintigraphic images showing hepatic ^99^mTc-pyrophosphate (^99^mTc-PYP) uptake in the experimental groups. Compared with the control and vitamin D–treated groups, markedly increased hepatic ^99^mTc-PYP uptake was observed in the doxorubicin (DOX) group. Co-administration of vitamin D (5000 or 60,000 IU/kg) with doxorubicin reduced hepatic tracer uptake relative to DOX alone, although uptake remained higher than in controls. Images are representative of each experimental group. In the hepatic region, regions of interest (ROIs) were delineated to quantify radiotracer activity. Increased hepatic uptake indicates abnormal tracer retention, whereas activity observed in the kidneys and urinary bladder reflects physiological radiopharmaceutical excretion. L: liver region of interest (ROI); RK: right kidney; LK: left kidney; B: urinary bladder. Tracer accumulation in the kidneys and bladder reflects physiological renal excretion.

**Figure 3:**
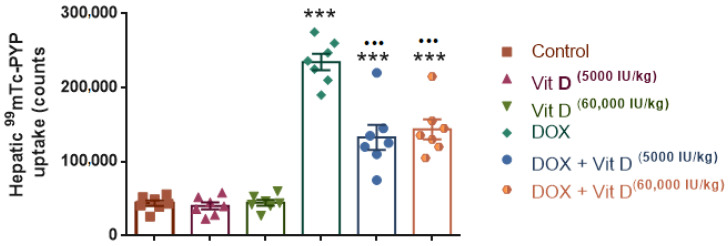
Quantitative analysis of hepatic ^99^mTc-pyrophosphate (^99^mTc-PYP) uptake across the experimental groups. Hepatic ^99^mTc-PYP uptake was markedly increased in the doxorubicin (DOX) group compared with the control group. Co-administration of vitamin D (5000 or 60,000 IU/kg) significantly reduced tracer uptake relative to DOX alone, although uptake levels remained higher than those observed in controls. Data are presented as mean ± SEM. *** *p* < 0.001 vs. control; ^•••^ *p* < 0.001 vs. DOX.

Hepatic ^99^mTc-PYP uptake differed significantly among the experimental groups (F(5,36) = 57.97, *p* < 0.0001, R^2^ = 0.8895). Doxorubicin administration resulted in a marked increase in ^99^mTc-PYP uptake compared with the control group (*p* < 0.0001), whereas vitamin D treatment alone (5000 or 60,000 IU/kg) did not significantly affect uptake relative to controls (*p* = 0.9999 and *p* > 0.9999, respectively). In contrast, co-administration of vitamin D with doxorubicin significantly reduced ^99^mTc-PYP uptake compared with DOX alone (both doses *p* < 0.0001). Compared with the control group, ^99^mTc-PYP uptake remained significantly elevated in the DOX + vitamin D-treated groups (*p* < 0.0001), while no statistically significant difference was observed between the two vitamin D doses within the DOX-treated groups (*p* = 0.9756) (Figure 2 and Figure 3).

### 3.3. Markers of Liver Injury and Hepatocellular Damage

#### 3.3.1. Serum Liver Enzyme Markers (AST and ALT)

Serum aspartate aminotransferase (AST) and alanine aminotransferase (ALT) levels differed significantly among the experimental groups (AST: F(5,36) = 34.83, *p* < 0.0001, R^2^ = 0.8287; ALT: F(5,36) = 59.30, *p* < 0.0001, R^2^ = 0.8917). Doxorubicin administration resulted in a marked increase in both AST and ALT levels compared with the control group (all *p* < 0.0001), whereas vitamin D treatment alone (5000 or 60,000 IU/kg) did not significantly affect either enzyme relative to controls (all *p* > 0.05). In contrast, co-administration of vitamin D with doxorubicin significantly reduced AST and ALT levels compared with DOX alone (ALT: both doses *p* < 0.0001; AST: both doses *p* < 0.0001).

**Figure 4:**
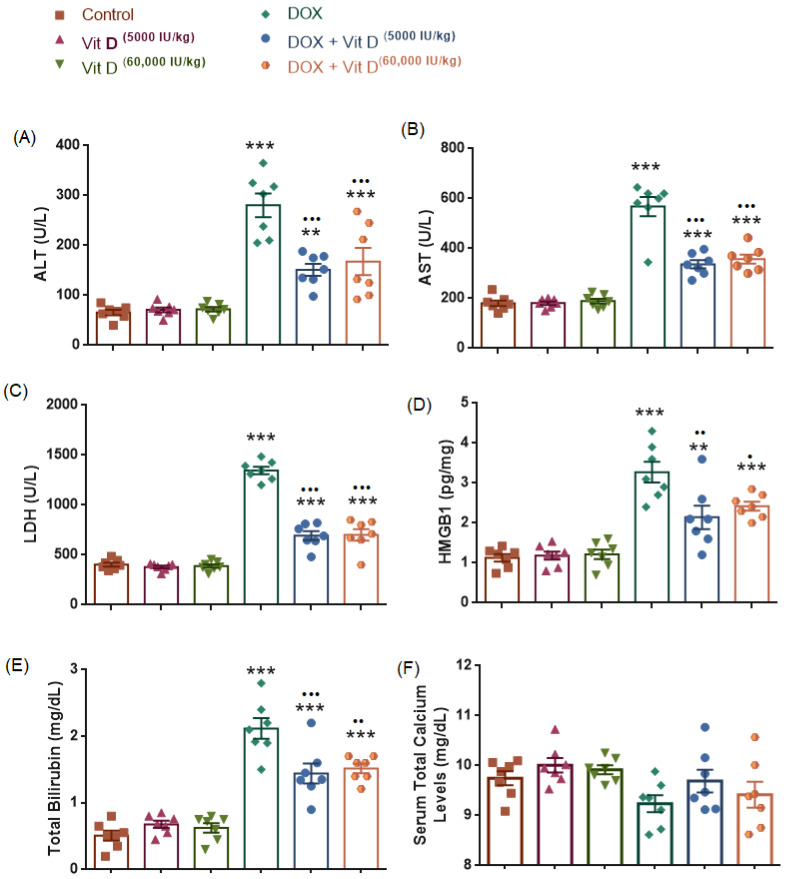
Effects of doxorubicin and vitamin D on serum liver injury markers and hepatic HMGB1. (**A**) Serum ALT, (**B**) AST, (**C**) LDH, (**D**) hepatic HMGB1, and (**E**) serum total bilirubin and (**F**) serum total calcium levels in control, vitamin D (5000 or 60,000 IU/kg), DOX, and DOX + vitamin D groups. DOX markedly increased all markers compared with controls, while vitamin D alone had no significant effect. Vitamin D co-administration significantly attenuated DOX-induced increases, although values generally remained higher than control levels. Data are presented as mean ± SEM. ** *p* < 0.01 *** *p* < 0.001 vs. control; ^•^ *p* < 0.05, ^••^ *p* < 0.01, ^•••^ *p* < 0.001 and vs. DOX.

Compared with the control group, AST levels remained significantly elevated in the DOX + vitamin D groups (DOX + Vit D 5000 IU/kg, *p* < 0.01; DOX + Vit D 60,000 IU/kg, *p* < 0.001). However, vitamin D co-administration significantly reduced AST levels compared with the DOX group (both *p* < 0.001). No statistically significant difference was observed between the two vitamin D doses within the DOX-treated groups for either ALT or AST (all *p* > 0.05) (Figure 4).

#### 3.3.2. Markers of Liver Injury and Damage-Associated Molecular Patterns (Serum LDH and Hepatic HMGB1)

As shown in Figure 4, serum lactate dehydrogenase (LDH) and hepatic high mobility group box 1 (HMGB1) levels differed significantly among the experimental groups (LDH: F(5,36) = 109.1, *p* < 0.0001, R^2^ = 0.9381; HMGB1: F(5,36) = 22.81, *p* < 0.0001, R^2^ = 0.7601). Doxorubicin administration resulted in a marked increase in both LDH and HMGB1 levels compared with the control group (all *p* < 0.0001). Vitamin D treatment alone (5000 or 60,000 IU/kg) did not significantly alter LDH or HMGB1 levels relative to controls (all *p* > 0.05).

In contrast, co-administration of vitamin D with doxorubicin significantly reduced LDH and HMGB1 levels compared with DOX alone (all *p* < 0.0001). Compared with the control group, LDH levels remained significantly elevated in the DOX + vitamin D groups (all *p* < 0.0001), whereas HMGB1 levels were significantly lower than DOX but still differed from controls (Control vs. DOX + Vit D 5000 IU/kg, *p* = 0.0007; Control vs. DOX + Vit D 60,000 IU/kg, *p* = 0.0030). No statistically significant difference was observed between the two vitamin D doses within the DOX-treated groups for either LDH or HMGB1 (*p* > 0.05).

#### 3.3.3. Total Bilirubin

Serum total bilirubin levels differed significantly among the experimental groups (F(5,36) = 38.21, *p* < 0.0001, R^2^ = 0.8414). Doxorubicin administration significantly increased total bilirubin levels compared with the control group (*p* < 0.0001), whereas vitamin D treatment alone (5000 or 60,000 IU/kg) did not significantly affect bilirubin levels relative to controls (*p* > 0.05). Co-administration of vitamin D with doxorubicin significantly reduced total bilirubin levels compared with DOX alone (*p* < 0.01). No significant difference was observed between the two vitamin D doses within the DOX-treated groups (*p* > 0.05) (Figure 4).

#### 3.3.4. Total Calcium

Although the overall ANOVA indicated a statistical difference among groups, post hoc Tukey analysis revealed no significant pairwise differences. However, Tukey’s multiple comparison test revealed no statistically significant pairwise differences between the control, vitamin D (5000 IU/kg), vitamin D (60,000 IU/kg), DOX, DOX + vitamin D (5000 IU/kg), and DOX + vitamin D (60,000 IU/kg) groups (all *p* > 0.05) (Figure 4).

### 3.4. Serum 25-Hydroxyvitamin D [25(OH)D] Levels

Serum 25-hydroxyvitamin D [25(OH)D] levels differed significantly among the six experimental groups (F(5,36) = 38.46, *p* < 0.0001, R^2^ = 0.842). Both vitamin D-treated groups exhibited significantly higher serum 25(OH)D levels compared with the control group (Vitamin D 5000 IU/kg and 60,000 IU/kg; both *p* < 0.0001). In contrast, the DOX-treated group did not differ significantly from controls (*p* = 0.9863), indicating that doxorubicin administration alone did not alter circulating vitamin D levels.

**Figure 5:**
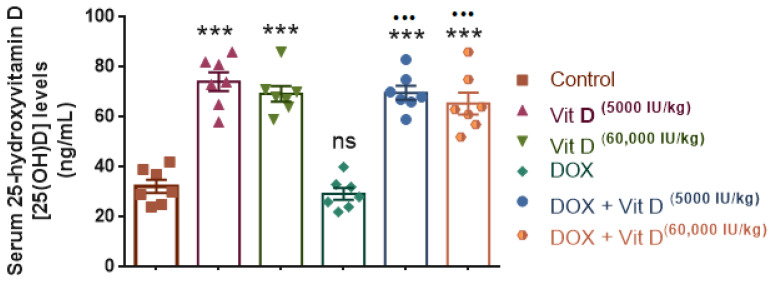
Serum 25-hydroxyvitamin D [25(OH)D] levels across the experimental groups. Vitamin D supplementation (5000 or 60,000 IU/kg) significantly increased serum 25-hydroxyvitamin D [25(OH)D] levels compared with the control group, whereas doxorubicin (DOX) alone did not significantly alter circulating 25(OH)D levels. Co-administration of vitamin D with doxorubicin resulted in significantly higher 25(OH)D levels compared with the DOX group. Data are presented as mean ± SEM. *** *p* < 0.001 vs. control; ^•••^ *p* < 0.001 vs. DOX; ns, not significant.

Co-administration of vitamin D with doxorubicin resulted in a marked increase in serum 25(OH)D concentrations compared with the DOX group alone (DOX + vitamin D 5000 IU/kg and DOX + vitamin D 60,000 IU/kg; both *p* < 0.0001). However, no statistically significant difference was observed between the two vitamin D doses within the DOX-treated groups (*p* > 0.05), indicating that increasing the vitamin D dose did not confer an additional rise in serum 25(OH)D levels under the present experimental conditions (Figure 5).

### 3.5. Biochemical Markers of Oxidative and Antioxidant Status

#### 3.5.1. Malondialdehyde (MDA) Levels

**Figure 6:**
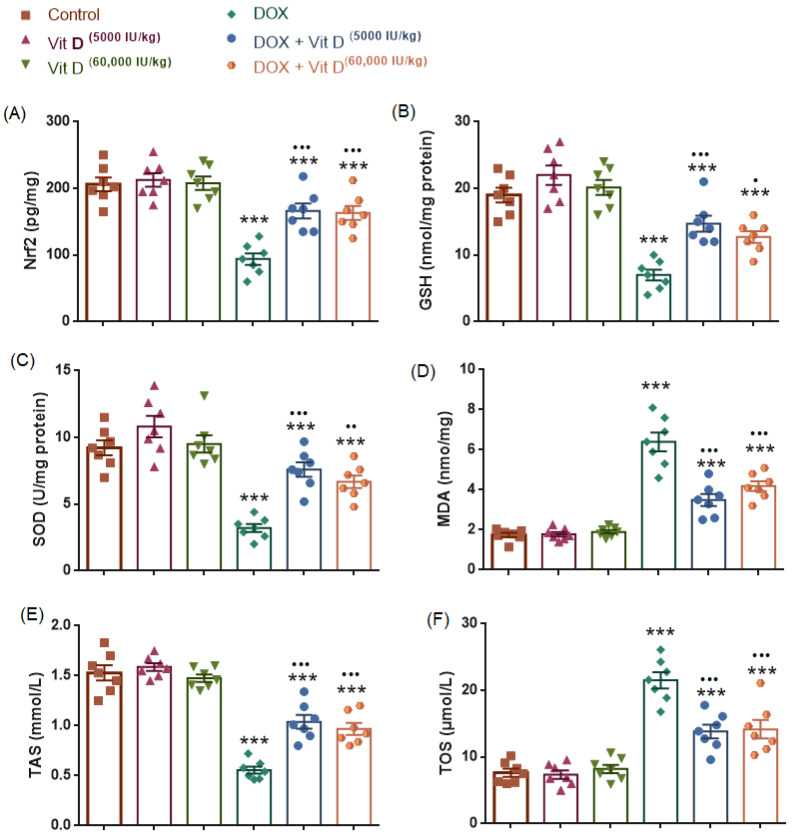
Effects of doxorubicin and vitamin D on hepatic oxidative stress markers. (**A**) Nrf2, (**B**) GSH, (**C**) SOD, (**D**) MDA, (**E**) TAS, and (**F**) TOS levels in control, vitamin D (5000 or 60,000 IU/kg), DOX, and DOX + vitamin D groups. Doxorubicin markedly impaired antioxidant defenses and increased oxidative stress, whereas vitamin D co-administration significantly attenuated these alterations. Data are presented as mean ± SEM. *** *p* < 0.001 vs. control; ^•^ *p* < 0.05, ^••^ *p* < 0.01, ^•••^ *p* < 0.001 and vs. DOX.

Shown in Figure 6, Hepatic MDA levels differed significantly among the experimental groups (F(5,36) = 51.70, *p* < 0.0001, R^2^ = 0.8778). Doxorubicin administration resulted in a significant increase in MDA levels compared with the control group (*p* < 0.0001), whereas vitamin D treatment alone (5000 or 60,000 IU/kg) did not significantly affect MDA levels (*p* > 0.9999 and *p* = 0.9979, respectively). Compared with the DOX group, both vitamin D doses administered alone were associated with significantly lower MDA levels (Vit D 5000 IU/kg vs. DOX and Vit D 60,000 IU/kg vs. DOX; both *p* < 0.0001). Co-administration of vitamin D with doxorubicin significantly reduced MDA levels relative to DOX alone (DOX + Vit D 5000 IU/kg, *p* < 0.001; DOX + Vit D 60,000 IU/kg, *p* < 0.0001). No statistically significant difference was observed between the two vitamin D doses within the DOX-treated groups (*p* = 0.4048).

#### 3.5.2. Nuclear Factor Erythroid 2–Related Factor 2 (Nrf2) Levels

Hepatic Nrf2 levels differed significantly among the experimental groups (F(5,36) = 19.79, *p* < 0.0001, R^2^ = 0.7332). Nrf2 levels were significantly decreased in the DOX group compared with controls (*p* < 0.0001). Vitamin D administration alone (5000 or 60,000 IU/kg) did not significantly alter Nrf2 levels compared with the control group (*p* = 0.9976 and *p* > 0.9999, respectively). Co-administration of vitamin D with doxorubicin significantly increased Nrf2 levels relative to DOX alone (DOX + Vit D 5000 IU/kg, *p* = 0.0002; DOX + Vit D 60,000 IU/kg, *p* = 0.0004). No significant difference was observed between the two vitamin D doses within the DOX-treated groups (*p* > 0.9999), nor between the two vitamin D doses administered alone (*p* = 0.9994) (Figure 6).

#### 3.5.3. Glutathione (GSH) Levels and Superoxide Dismutase (SOD) Activity

GSH levels and SOD activity levels in liver tissue differed significantly across the experimental groups (GSH: F(5,36) = 24.84, *p* < 0.0001, R^2^ = 0.7753; SOD: F(5,36) = 22.41, *p* < 0.0001, R^2^ = 0.7569). Doxorubicin administration resulted in a statistically significant reduction in both GSH and SOD levels compared with the control group (GSH: *p* < 0.0001; SOD: *p* < 0.0001). Vitamin D treatment alone (5000 or 60,000 IU/kg) did not significantly alter GSH or SOD levels relative to controls (GSH: *p* = 0.4214 and *p* = 0.9780; SOD: *p* = 0.3823 and *p* = 0.9991, respectively). Co-administration of vitamin D with doxorubicin led to a significant increase in GSH and SOD levels relative to DOX alone (GSH: *p* < 0.001 and *p* = 0.0110; SOD: *p* < 0.0001 and *p* = 0.0015, for 5000 and 60,000 IU/kg, respectively). No statistically significant difference was observed between the two vitamin D doses within the DOX-treated groups for either parameter (GSH: *p* = 0.8023; SOD: *p* = 0.8647) (Figure 6).

#### 3.5.4. Total Antioxidant Status (TAS) and Total Oxidant Status (TOS)

Total antioxidant status (TAS) and total oxidant status (TOS) differed significantly among the experimental groups (TAS: F(5,36) = 53.83, *p* < 0.0001, R^2^ = 0.8820; TOS: F(5,36) = 32.78, *p* < 0.0001, R^2^ = 0.8199). Doxorubicin administration resulted in a statistically significant decrease in total antioxidant status (TAS) and a concomitant increase in total oxidant status (TOS) compared with the control group (TAS: *p* < 0.0001; TOS: *p* < 0.0001). Vitamin D treatment alone (5000 or 60,000 IU/kg) did not significantly alter TAS or TOS levels relative to controls (TAS: *p* = 0.9771 and *p* = 0.9817; TOS: *p* > 0.9999 and *p* = 0.9984, respectively). Co-administration of vitamin D with doxorubicin significantly improved redox status, as indicated by increased TAS levels and reduced TOS levels compared with the DOX group (TAS: *p* < 0.0001 for both doses; TOS: *p* < 0.0001 for both doses). No statistically significant difference was observed between the two vitamin D doses within the DOX-treated groups for either TAS (*p* = 0.9414) or TOS (*p* = 0.9999) (Figure 6).

### 3.6. Inflammatory and Anti-Inflammatory Cytokines

#### 3.6.1. Effects of Doxorubicin and Vitamin D on Pro-Inflammatory Cytokines (TNF-α, IL-6, and IL-1β)

**Figure 7:**
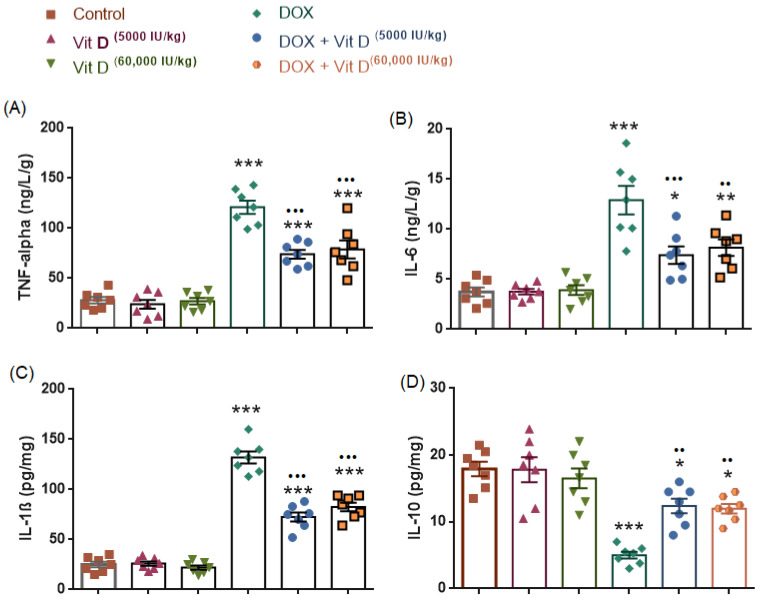
Effects of doxorubicin and vitamin D on inflammatory cytokine levels. (**A**) TNF-α, (**B**) IL-6, (**C**) IL-1β, and (**D**) IL-10 levels in control, vitamin D (5000 or 60,000 IU/kg), doxorubicin (DOX), and DOX + vitamin D–treated groups. Doxorubicin markedly increased pro-inflammatory cytokines (TNF-α, IL-6, and IL-1β) and reduced IL-10 levels compared with controls, whereas vitamin D treatment alone had no significant effect. Co-administration of vitamin D significantly attenuated DOX-induced inflammatory responses and partially increased IL-10 levels. Data are presented as mean ± SEM. * *p* < 0.05, ** *p* < 0.01, *** *p* < 0.001 vs. control; ^••^ *p* < 0.01, ^•••^ *p* < 0.001 vs. DOX.

As shown in Figure 7, hepatic tumor necrosis factor-α (TNF-α), interleukin-6 (IL-6), and interleukin-1β (IL-1β) levels differed significantly among the experimental groups (TNF-α: F(5,36) = 50.18, *p* < 0.0001, R^2^ = 0.8745; IL-6: F(5,36) = 19.95, *p* < 0.0001, R^2^ = 0.7348; IL-1β: F(5,36) = 126, *p* < 0.0001, R^2^ = 0.9461). Doxorubicin administration resulted in a significant increase in TNF-α, IL-6, and IL-1β levels compared with the control group (all *p* < 0.0001). Vitamin D treatment alone (5000 or 60,000 IU/kg) did not significantly alter TNF-α, IL-6, or IL-1β levels relative to controls (*p* > 0.05). In contrast, co-administration of vitamin D with doxorubicin significantly reduced cytokine levels compared with DOX alone, including TNF-α (both doses *p* < 0.0001), IL-6 (*p* = 0.0003 and *p* = 0.0015), and IL-1β (both doses *p* < 0.0001). No statistically significant difference was observed between the two vitamin D doses within the DOX-treated groups for TNF-α (*p* = 0.9887), IL-6 (*p* = 0.9917), or IL-1β (*p* = 0.6210) (Figure 7).

#### 3.6.2. Anti-Inflammatory Cytokine (IL-10)

As shown in Figure 7, hepatic interleukin-10 (IL-10) levels differed significantly among the experimental groups (F(5,36) = 16.73, *p* < 0.0001, R^2^ = 0.6991). Doxorubicin administration resulted in a significant decrease in IL-10 levels compared with the control group (*p* < 0.0001), indicating suppression of the anti-inflammatory response. Vitamin D treatment alone (5000 or 60,000 IU/kg) did not significantly alter IL-10 levels relative to controls (*p* > 0.9999 and *p* = 0.9593, respectively). In contrast, IL-10 levels remained significantly lower in the DOX + vitamin D-treated groups compared with the control group (Control vs. DOX + Vit D 5000 IU/kg, *p* = 0.0286; Control vs. DOX + Vit D 60,000 IU/kg, *p* = 0.0158). Compared with DOX alone, co-administration of vitamin D significantly increased IL-10 levels (DOX vs. DOX + Vit D 5000 IU/kg, *p* = 0.0016; DOX vs. DOX + Vit D 60,000 IU/kg, *p* = 0.0032). No statistically significant difference was observed between the two vitamin D doses within the DOX-treated groups (*p* = 0.9999).

### 3.7. Renal Function Markers (BUN and Creatinine)

**Figure 8:**
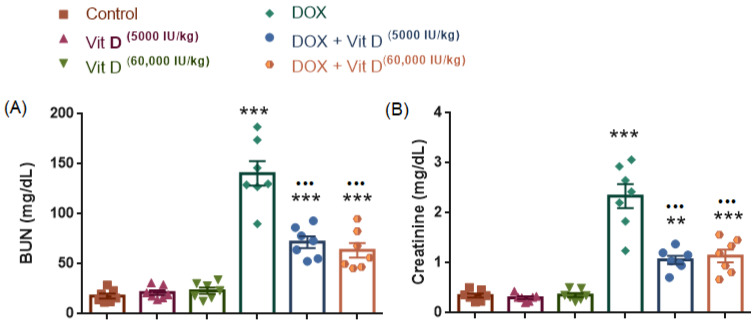
Effects of vitamin D on renal function markers in doxorubicin-treated rats. (**A**) Serum blood urea nitrogen (BUN) levels. (**B**) Serum creatinine levels. Doxorubicin (DOX) administration significantly increased both BUN and creatinine levels compared with the control group. Co-administration of vitamin D (5000 IU/kg or 60,000 IU/kg) significantly attenuated DOX-induced elevations in these renal injury markers. Vitamin D administration alone did not significantly alter BUN or creatinine levels compared with the control group. Data are presented as mean ± SEM (*n* = 7 per group). Statistical analysis was performed using one-way ANOVA followed by Tukey’s multiple comparisons test. ** *p* < 0.01, *** *p* < 0.001 vs. Control; ^•••^ *p* < 0.001 vs. DOX.

As shown in Figure 8, serum blood urea nitrogen (BUN) and creatinine levels differed significantly among the experimental groups (BUN: F(5,36) = 52.27, *p* < 0.0001, R^2^ = 0.8789; creatinine: F(5,36) = 43.51, *p* < 0.0001, R^2^ = 0.8580). Doxorubicin administration resulted in a marked increase in both BUN and creatinine levels compared with the control group (both *p* < 0.0001), indicating renal injury. Vitamin D treatment alone (5000 or 60,000 IU/kg) did not significantly alter BUN or creatinine levels relative to the control group (all *p* > 0.05). In contrast, co-administration of vitamin D with doxorubicin significantly reduced both BUN and creatinine levels compared with the DOX group (DOX + Vit D 5000 IU/kg: *p* < 0.0001 for both markers; DOX + Vit D 60,000 IU/kg: *p* < 0.0001 for both markers).

No statistically significant difference was observed between the two vitamin D doses within the DOX-treated groups for either BUN (*p* = 0.9468) or creatinine (*p* = 0.9970).

## 4. Discussion

The present study demonstrated that doxorubicin (DOX) markedly increased hepatic ^99^mTc-pyrophosphate (PYP) uptake and significantly elevated serum AST, ALT, LDH, total bilirubin, and hepatic HMGB1, indicating acute, severe hepatocellular injury. These findings align with previous experimental studies showing that DOX-induced hepatotoxicity is characterized by pronounced elevations in transaminase and LDH levels, reflecting extensive hepatocyte damage [23,24,25].

The current results indicate that the substantial rise in serum LDH after DOX administration reflects predominantly necrotic rather than apoptotic hepatocellular injury. LDH is rapidly released upon plasma membrane disruption and is widely recognized as a hallmark of acute toxic hepatonecrosis [25]. Under necrotic conditions, intracellular calcium overload and calcium–phosphate microprecipitate formation occur within damaged hepatocytes, creating high-affinity binding sites for bone-avid radiotracers such as ^99^mTc-PYP (19). Accordingly, the increased hepatic PYP uptake observed in the present study likely reflects necrosis-associated calcium deposition rather than nonspecific tracer accumulation. ^99^mTc-PYP uptake in our DOX-treated model aligns with earlier case reports demonstrating abnormal hepatic accumulation of bone-seeking radiotracers during acute liver necrosis. Lyons et al. (19) reported intense ^99^mTc-PYP uptake in massive hepatic necrosis. Likewise, Whitten and Luke [26] observed diffuse liver accumulation in a case of cocaine-induced toxic necrosis. Parker and Burke [27] described prominent hepatic ^99^mTc-MDP retention in Budd–Chiari syndrome, attributed to ischemic liver injury. These reports support the idea that necrotic hepatocytes selectively retain such tracers. Our DOX model reproduces this pattern, providing both biochemical and imaging evidence of acute necrotic liver injury.

In the present study, hepatic HMGB1 levels increased moderately, in contrast to the marked elevation in LDH after acute doxorubicin (DOX) administration. HMGB1 is a nuclear structural protein that functions as a danger-associated molecular pattern (DAMP) when released into the extracellular space upon cellular damage, particularly necrosis [28,29]. Experimental findings indicate that DOX induces a significant in-crease in hepatic HMGB1 expression, with studies reporting approximately a 2- to 3-fold increase relative to control levels [30]. This upregulation is linked to DOX-induced hepatocellular injury and necrosis, which facilitate the passive release of HMGB1 from damaged hepatocytes. Accordingly, the DOX-associated increase in HMGB1 observed in our study may reflect necrosis-associated inflammatory signaling. HMGB1 has been reported to interact with pattern-recognition receptors such as TLR4 and activate downstream NF-κB pathways, thereby contributing to pro-inflammatory cytokine production [28,30,31]. In line with this, elevated HMGB1 levels in our model may be associated with the enhanced inflammatory milieu. Notably, vitamin D co-administration significantly reduced hepatic HMGB1 compared with DOX alone, which may be consistent with attenuation of DAMP-related inflammatory signaling.

Many studies have reported that doxorubicin (DOX) induces oxidative stress through ROS production and Nrf2 downregulation [32,33,34,35]. In our study, DOX-treated rats showed marked increases in hepatic MDA and TOS, indicating enhanced lipid peroxidation and oxidant burden. These changes were accompanied by significant decreases in Nrf2, GSH, SOD, and TAS, supporting depletion of antioxidant defenses. Consistent with previous findings, hepatic Nrf2 levels were significantly reduced in the DOX group, indicating impaired activation of the antioxidant response [36,37,38]. In the present study, Nrf2 levels were measured as total protein in liver homogenates using ELISA. Therefore, conclusions regarding nuclear translocation or transcriptional activation of downstream targets such as HO-1 and NQO1 cannot be definitively drawn [39]. The observed increase in Nrf2 following vitamin D treatment may reflect either direct modulation of the pathway or normalization of a compensatory response secondary to reduced oxidative stress. Further studies involving nuclear-cytoplasmic fractionation and target gene validation are required to clarify this mechanism [33].

Importantly, loss of redox balance was reflected in elevated serum AST, ALT, and LDH, suggesting membrane damage and hepatocellular leakage. These biochemical alterations align with the mechanism of DOX-induced oxidative hepatotoxicity and confirm that oxidative stress plays a central role in liver injury. These findings suggest an association with impaired Nrf2-related antioxidant signaling, while elevated MDA and enzyme leakage (AST/ALT/LDH) provide functional evidence of structural membrane injury.

Growing evidence indicates that oxidative stress and inflammation are closely linked in doxorubicin (DOX)-induced liver injury. ROS generated by DOX can activate NF-κB, increasing expression of pro-inflammatory cytokines such as TNF-α, IL-6, and IL-1β, while suppressing anti-inflammatory cytokines like IL-10 [34,35,40]. Consistent with these reports, our findings showed elevated hepatic levels of TNF-α, IL-6, and IL-1β, along with reduced IL-10 in DOX-treated rats.

Notably, our findings showed that vitamin D exerted a protective effect by modulating oxidative stress and inflammation in DOX-induced liver injury. Previous studies report that active vitamin D (calcitriol) activates the Keap1–Nrf2 pathway, which boosts antioxidant defenses such as GSH and SOD and helps reduce ROS-induced damage [41,42,43]. In addition, vitamin D suppresses inflammatory pathways by inhibiting NF-κB and decreasing the production of pro-inflammatory cytokines such as TNF-α, IL-6, and IL-1β, while increasing IL-10 levels. These combined effects help rebalance the inflammatory environment in the liver and reduce tissue injury. Together, these antioxidant and anti-inflammatory actions of vitamin D may explain its protective role against DOX-induced liver damage.

The current study demonstrates that vitamin D administration significantly increased serum 25-hydroxyvitamin D [25(OH)D] levels, whereas doxorubicin (DOX) alone did not significantly alter circulating 25(OH)D concentrations compared with the control group. Interestingly, both the single high-dose bolus and the repeated low-dose regimens resulted in comparable 25(OH)D levels. Vitamin D was administered as cholecalciferol (vitamin D3), an inactive precursor that undergoes hepatic 25-hydroxylation to form 25(OH)D and is subsequently converted in the kidney to its biologically active form, 1,25(OH)_2_D [44]. The active metabolite 1,25(OH)_2_D exerts its biological effects by binding to the vitamin D receptor (VDR), which regulates transcription of genes involved in oxidative stress and inflammatory signaling, and has been reported to suppress NF-κB activation while enhancing antioxidant responses through cross-talk with the Nrf2 pathway [44]. These mechanisms may partly explain the protective effects of vitamin D observed in the present study, including the attenuation of oxidative stress and inflammatory responses following DOX administration.

In our study, serum 25(OH)D levels in the vitamin D-treated groups remained comparable to the vitamin D group. Importantly, however, we did not quantify the biologically active metabolite 1,25(OH)2D or downstream catabolic metabolites; therefore, the present data cannot determine whether renal activation (via CYP27B1) or vitamin D catabolism was altered, nor can they confirm VDR activation in target tissues. The comparable 25(OH)D concentrations observed after bolus and repeated dosing may also reflect the fat-soluble nature of vitamin D, which allows storage in adipose tissue and gradual release into the circulation [45,46]. Moreover, the estimated dietary vitamin D intake from the standard chow was minimal compared with the pharmacological vitamin D doses administered in the experimental groups. Baseline serum 25-hydroxyvitamin D [25(OH)D] levels were not measured prior to DOX administration; therefore, further studies are required to clarify this relationship and to better understand the pharmacokinetic differences between bolus and repeated vitamin D administration in this experimental context.

A further noteworthy finding is the marked improvement in renal injury markers (BUN and creatinine) in the vitamin D co-treated groups compared with DOX alone. 25(OH)D requires 1α-hydroxylation by CYP27B1, classically in the kidney, to generate the active hormone 1,25(OH)2D. Attenuation of DOX-associated renal injury may therefore be associated with preservation of renal vitamin D activation capacity, potentially influencing downstream VDR signaling [47,48]. Emerging evidence also indicates that, when sufficient 25(OH)D substrate is available, 1,25(OH)2D can be produced in non-renal tissues under specific physiological and inflammatory conditions [49,50]. Neither CYP27B1 expression nor circulating (or tissue) 1,25(OH)2D was measured in the present study. These interpretations should therefore be considered mechanistic hypotheses requiring further experimental validation.

In the present study, serum calcium levels showed a slight, non-significant decrease in the DOX-treated group compared with controls. However, serum total calcium did not differ between groups at the terminal time point, indicating that these changes were not statistically or biologically significant under the present experimental conditions. Serum calcium responses to DOX have been reported to vary across experimental settings, although alterations are often minimal in acute or short-term models [47,51,52,53]. In line with this, the absence of significant differences in our study suggests that neither DOX nor the applied cholecalciferol regimens induced overt disturbances in systemic calcium levels. However, because only total calcium was measured at a single time point (without ionized calcium, phosphate, PTH/FGF23, or histological assessment of soft-tissue calcification), transient perturbations in mineral homeostasis or subclinical ectopic calcification cannot be excluded. Accordingly, the absence of hypercalcemia should be interpreted as a limited safety indicator rather than definitive evidence of systemic safety.

High-dose vitamin D administration may raise concerns regarding hypercalcemia and soft-tissue calcification. However, the dose used in the present study (60,000 IU/kg; ≈1.5 mg/kg cholecalciferol) is markedly lower than doses reported to cause lethal toxicity in rodents (~40–50 mg/kg) [54]. Several experimental studies have demonstrated protective effects of similar vitamin D doses without systemic toxicity [22,55,56]. Consistent with these findings, serum calcium levels remained unchanged in our study following vitamin D administration, indicating the absence of vitamin D-induced hypercalcemia. Previous experimental and clinical studies also suggest that hypercalcemia after high-dose cholecalciferol administration is uncommon and depends on dosing regimen, route of administration, and sampling time points [44,46,57,58]. In agreement with these reports, both the repeated 5000 IU/kg regimen and the single 60,000 IU/kg oral dose in our study significantly increased serum 25(OH)D levels without altering circulating calcium concentrations.

In the present study, doxorubicin (DOX) exposure was accompanied by a modest downward shift in the body-weight trajectory, while baseline and terminal body weights remained comparable across groups. This subtle pattern is consistent with early-phase anthracycline-related growth suppression, whereas more pronounced weight loss typically emerges with longer, cumulative dosing in rats and can become evident from approximately the third week onward in chronic protocols [59,60,61]. Mechanistically, DOX can impair skeletal-muscle homeostasis through mitochondrial dysfunction and reactive oxygen species generation, with activation of proteolytic programs including the ubiquitin–proteasome system and autophagy [62]. Reduced energy intake may further contribute, as rodent DOX regimens have been associated with anorexia and decreased caloric intake, potentially compounded by chemotherapy-related gastrointestinal symptoms and inflammation-linked dysregulation of appetite signaling [63,64,65]. In our acute, three-day DOX protocol, vitamin D co-administration showed an apparent but statistically non-significant trend toward preserving body weight. Although the vitamin D/VDR axis has been implicated in skeletal-muscle oxidative metabolism and mitochondrial function, these mechanisms were not directly evaluated in the current study [66,67]. Therefore, any interpretation of vitamin D as attenuating DOX-associated anorexia/cachexia should be made cautiously, and longer-term studies incorporating food intake and body composition endpoints are warranted.

These findings underscore the translational relevance of our results. Vitamin D supplementation-whether administered daily or as a single bolus-significantly increased serum 25-hydroxyvitamin D [25(OH)D] levels in rats, with no significant difference between the two dosing strategies. These findings suggest that vitamin D supplementation may have a protective role against liver injury in the context of chemotherapy-induced hepatotoxicity. Although ^99^mTc-PYP scintigraphy typically shows minimal hepatic uptake under physiological conditions, our results suggest that pathological uptake may occur in settings of acute drug-induced liver damage. Such uptake may therefore serve as a non-invasive indicator of hepatocellular injury and could aid in the early detection or assessment of hepatic damage.

An additional question raised by the present findings is whether pre-existing vitamin D deficiency could exacerbate DOX-induced liver injury. Previous studies indicate that vitamin D deficiency is associated with increased oxidative stress and pro-inflammatory signaling in experimental liver injury models, whereas activation of the vitamin D/vitamin D receptor (VDR) axis may attenuate hepatic inflammation and fibrogenic responses [68,69]. Similarly, DOX-induced hepatotoxicity has been reported to involve excessive reactive oxygen species generation, lipid peroxidation, and downstream inflammatory cascades [35,70]. These observations suggest that reduced vitamin D status may diminish hepatic resilience and potentially amplify DOX-associated injury. This issue is translationally relevant, as low serum 25(OH)D concentrations are frequently reported in cancer patients undergoing chemotherapy [71,72], and a high prevalence of vitamin D deficiency has been documented in oncology settings more broadly [73]. Additionally, specific chemotherapeutic regimens such as those used in colorectal cancer have been linked to severe depletion of circulating vitamin D levels [74]. Future studies employing vitamin D-deficient diets and longitudinal monitoring of vitamin D metabolism are warranted to determine whether deficiency directly increases susceptibility to DOX-induced hepatotoxicity.

## 5. Conclusions

Vitamin D supplementation attenuated DOX-induced organ injury, as reflected by improvements in oxidative stress, inflammatory markers, and renal function parameters. Overall, we hypothesize that pharmacological cholecalciferol supplementation may confer protection by modulating oxidative stress and inflammatory responses through the vitamin D endocrine axis. This effect is most plausibly mediated by increased conversion of 25(OH)D to the active metabolite 1,25(OH)2D and subsequent activation of VDR-dependent signaling pathways. However, as neither 1,25(OH)2D levels nor VDR activation were directly measured, this mechanism should be interpreted as a biologically plausible hypothesis rather than a confirmed pathway. Future studies are warranted to directly assess CYP27B1 activity, circulating and tissue levels of 1,25(OH)2D, and VDR activation in order to clarify the mechanistic basis of these protective effects.

### Limitation

Several limitations should be considered when interpreting the present findings. First, histopathological evaluation of liver tissue was not included in the present study; therefore, structural hepatic alterations could not be directly assessed. Second, animals were maintained on a standard laboratory diet and were not rendered vitamin D deficient, and baseline serum 25-hydroxyvitamin D concentrations were not measured prior to intervention. In addition, Nrf2 was quantified as total protein using ELISA; therefore, nuclear translocation and transcriptional activation of the Nrf2 pathway could not be directly assessed. Future studies incorporating histological evaluation, vitamin D-deficient models, and cellular localization of Nrf2 would help further clarify the mechanisms underlying DOX-induced hepatotoxicity.

Additional limitations should be acknowledged regarding vitamin D assessment. Serum 25(OH)D was quantified using an ELISA-based immunoassay; although widely used, immunoassays can be susceptible to method-dependent bias, matrix effects, and cross-reactivity with structurally related metabolites, whereas LC-MS/MS provides higher analytical specificity and enables simultaneous discrimination of multiple vitamin D metabolites. Moreover, only 25(OH)D was measured; thus, potential changes in the active hormone 1,25(OH)2D and downstream catabolic metabolites (e.g., 24-hydroxylated products) could not be assessed. Future studies should therefore use LC–MS/MS-based panels to quantify 25(OH)D2/D3, 1,25(OH)2D, and key catabolic metabolites to more precisely define vitamin D activation and turnover in this model. Importantly, VDR activation was not directly evaluated in the present study, and the active mediator of these effects is likely to be 1,25(OH)2D rather than the supplemented 25(OH)D itself.

## Data Availability

The data that support the findings of this study are not publicly available due to ethical reasons but are available from the corresponding author upon request.
